# Patients’ perceptions of healthcare professionalism—a Romanian experience

**DOI:** 10.1186/s12913-017-2412-z

**Published:** 2017-07-06

**Authors:** Daniela Popa, Daniela Druguș, Florin Leașu, Doina Azoicăi, Angela Repanovici, Liliana Marcela Rogozea

**Affiliations:** 1 Department of Psychology and Training in Education, Transilvania University of Brasov, Str. Nicolae Balcescu no. 56, 500019 Brasov, Romania; 2 Department of Medicine, University of Medicine and Pharmacy, Iasi, Romania; 3 Department of Fundamental Disciplines and Clinical Prevention, Transilvania University of Brasov, Brasov, Romania; 4 Department of Product Design and Environment, Transilvania University of Brasov, Brasov, Romania

**Keywords:** Patient dissatisfaction, professionalism, types of healthcare services, services quality, ex-communist country

## Abstract

**Background:**

The main objective of this cross sectional study was to assess the psychometric properties of a new research instrument. The secondary aim was to analyze patients’ levels of dissatisfaction with the professionalism of medical staff.

**Methods:**

A social survey questionnaire was created and administered online. The instrument consisted of two scales: the 30-item patient dissatisfaction scale and the 10 items institutional scale. In this article, we assessed only the patient dissatisfaction scale. The research population includes 1838 subjects. The statistical procedures used were descriptive statistics, Pearson’s correlation, and factorial analyses with the SPSS.19 software. The internal consistency of the instrument was determined using the Cronbach’s alpha coefficient. We used a principal component analysis to investigate the factorial validity of the scale.

**Results:**

The patients’ scale of dissatisfaction obtained an alpha Cronbach score of 0.81. Three latent factors corresponding to three dimensions of dissatisfaction emerged from the data: medical staff’s ability to communicate, medical staff’s hygiene, as well as sanitary and privacy conditions within the hospital. The first factor explained 43.47% of the variance in patient dissatisfaction, the second factor explained 10.24%, and the third factor explained 7.59%; overall, the three factors explained 61.30% of the total variance.

**Conclusion:**

The Romanian healthcare system has an organization and management structure which has shown few changes since the communist period. Our study indicates that although more than 25 years have passed since the political regime changed in Romania and the introduction of a different system of social care, there have been no corresponding changes in the medical staff’s mentality or in the way that patients are approached. The present assessment of patient dissatisfaction is not a strictly theoretical exercise; it also represents a valuable instrument for healthcare system management.

**Electronic supplementary material:**

The online version of this article (doi:10.1186/s12913-017-2412-z) contains supplementary material, which is available to authorized users.

## Background

Patient satisfaction is, at least theoretically, an important objective of hospital managers, “a proxy, and an effective indicator, to measure the success of doctors and hospitals” [1]. It has become one of the most used criteria for establishing health care quality [2–4]. Previous studies argue that satisfied customers tend to develop trusting relationship with healthcare providers and to reveal higher levels of cooperation, which determine higher health outcomes [5, 6].

Customer satisfaction is an attitude based on the perception of service quality. Health care literature has described the following two dimensions of quality health services as the basic elements of patient satisfaction: procedural dimension (the anticipation of the patient’s need, treatment provided, and hospitalization and discharge methods) and personal dimension (the physical aspect of cleanness, maintenance, relationships with the medical staff) [7–12]. Furthermore, patient satisfaction comprises two aspects: (1) cognitive processes (understanding the received information, the necessary procedures) and (2) emotional reactions to the elements of the institution’s structure (human, materials, financial resources, and the hospital environment), the management process (technical and interpersonal factors), and the results of the medical care provided [13, 14].

One of the most used investigation methods of patient’ satisfaction level is questionnaire-based survey, which usually is applicable in the discharge moment from hospital. Nonetheless, it is questionable whether asking about the patient’s level of satisfaction upon leaving the hospital has become a routine procedure.

A literature review suggests that the satisfaction-measurement instruments tend to use broad and vague terms, which lead to short, superficial, and affirmative answers, without any real meaning [15, 16]. Some researchers have pointed out the ambiguity of the notion of “patient satisfaction” [17]. Although the term is used in “evaluating and directing the delivery of healthcare”, it is more closely related to the quality of services rather than the quality of care. Pomerantz has described various changes that have occurred in recent years in the way healthcare service users perceive the quality of such services [7] and has suggested that an increased focus on the patients’ needs and preferences may help better orient medical care, compared to the focus on the adherence to care standards.

One aspect of satisfaction and dissatisfaction is the health care providers’ professionalism. Professionalism is one large area including many aspects such as: up to date medical knowledge, high level of development of clinical competences, strong ethical principles and standards, sharing values as respect and honesty, and empathetic, courteous, and kind attitude in interaction with the patients [18, 19]. In economically developed countries of Western Europe and the US, the orientation of the medical act has changed from a predominantly doctor-oriented approach to a patient-centered culture. Not the same can be said about Eastern Europe, which is dominated by corruption, informal payments and the need to reform [5, 20]. Eastern European countries have fallen behind Western European countries in terms of health care quality since the 1970s, because of the lingering influence of communist policy throughout the region [21].

Patient satisfaction in Romania is among the lowest in Europe [22]. Romania faces another problem, which is related to the access of unprivileged communities to health services [23]. In Romania, the general dissatisfaction and pessimism owed to the country’s poor economic performance and limitations in individual freedom may explain—to a greater degree than the behavioral or environmental factors—the deterioration of the state of health throughout the country [24, 25]. Often, Romanian patients only arrive at the hospital when their health status is already seriously poor. The most frequently given reasons for this are the low economic status, not making health a priority for financial reasons, and the way people are treated in Romanian hospitals.

Notably, there is little research explicitly focused on the patients’ levels of dissatisfaction in Romania. In previous studies, the greatest factors that increase frustration among patients have been: the quality of the hospital environment, of the food received in hospital, the aspect of cleanness, maintenance, the communication patient-doctor [26–28]. The areas in need of improvement so as to ensure the quality of Romanian healthcare services are: implementation of strategies in ergonomics, equipment, technology, employees’ appearance [29].

In the present study, we have examined patient satisfaction regarding the health care providers’ professionalism of in Romanian hospitals. We have analyzed the causes of patients’ dissatisfaction in Romania, the relative importance of these causes to patients, the problems that patients face during hospitalization. In order to attain the above-mentioned aims, a tool to measure patient dissatisfaction was developed.

## Methods

This study was conducted between January and May 2014. Its main objective was to assess the psychometric properties of a new research instrument. The secondary objective of the study was to identify the patients’ levels of dissatisfaction with the medical staff’s professionalism. The ethics committee of the University of Medicine and Pharmacy in Iasi approved all aspects of the study. The questionnaire was filled in anonymously. Respondents gave their written consent as a part of participating in the survey. Furthermore, the respondents were informed that they could withdraw their participation anytime during questionnaire filling in. We informed them that filling in the questionnaire was considered indicative of their agreement with the research terms and their understanding of their rights as participants. No minors or children were enrolled in the study.

A social survey questionnaire was created and administered online using SurveyMonkey [30]. The questionnaire was created through qualitative research with eight focus groups on the most important aspects of patient dissatisfaction. A copy of this instrument can be found in Additional file 1, under the name “Patient satisfaction assessment questionnaire”. Agreements were signed with several medical centres across the country to transmit the questionnaire through the online medium to patients discharged the previous month. In order to identify potential respondents, the hospitals’ databases were used. The questionnaires were sent to former patients in order to be filled, immediately after discharge. The criterion for the selection of the respondents was their having been hospitalized for at least 3 days. All respondents who filled in the questionnaire in full were accepted to participate in this study.

The questionnaire included a brief introduction stating the purpose of the research, a few instructions for completing the questionnaire, and the respondents’ rights as participants. Demographic data were also collected, and these included the participants’ sex (male or female), age and education level. In addition, the diagnosis and number of hospitalization days were used to determine the influence on the criterion variable.

The questionnaire addressed factors related to the hospitalization experience that can generate patient dissatisfaction. After analyzing the data obtained from the previously mentioned focus groups, 3 key factors stood out as the sources of dissatisfaction among patients. The first main factor identified was the medical staff’s ability to communicate. It includes 3 items that refers to the explanation of procedures and treatments, one’s being informed about scheduled procedures, and the self-introduction of the medical staff members. The second factor identified refers to the patient’s comfort, to wit if the patient was respected and how well (s)he was treated. This dimension contains 4 items that describe: the awareness of specific hospital smells, if the patients were placed in a mixed ward (containing both men and women), if they had feelings of thirst and hunger, and if they felt that they were being treated as an object rather than as a human being. The third factor refers to the physical and psychological discomfort. It represents the largest part of the questionnaire. It contains 23 items describing situations like: lights being turned on all the time, noise, medical staff talking about patients behind their backs, thermal comfort, difficulties in falling asleep, medical staff waking the patient up suddenly, tubes in the nose and mouth, pain experienced, lack of privacy, feelings of disorientation, fear of death and transmissible diseases, inability to communicate, insufficient contact with family and friends, feeling that nurses focus more on devices than on the patient, the medical staff uses unknown words, one being treated for by unknown doctors, and one’s consent for the treatment is not obtained.

### Description of the population

In order to analyze the patients’ levels of dissatisfaction with healthcare services, 1838 subjects were questioned. Gorsuch (1997) suggested that the minimum acceptable number of respondents is 10 per variable [31]. This was merely a guideline that we exceeded. The study group was chosen to ensure that the target group’s views were represented as accurately as possible.

Table 1 shows the distributions of demographic variables. We divided the education level into the following categories: elementary school, high school, college, university, and postgraduate studies. The study group consisted of 1070 (58.2%) female participants and 768 (41.8%) male participants. In terms of age, the patients showed a varied distribution. The smallest age group was the over 70 years old group (2.9%), while the largest were the age groups of 50–60 and 60–70 years (28.2% and 29.5%, respectively, 57.7% altogether). In terms of education, the smallest education group was the college education group (9.9%), while the largest were the education groups of university graduates (40.6%). As regards the number of hospitalization days, most respondents (910; 49.5%) had been hospitalized for 3 days, 361 (19.6%) for 7 days, 368 (20.0%) for 14 days, and only 199 respondents (10.8%) for the maximum hospitalization period of 21 days.

**Table 1:** Distributions of demographic variables

		Frequency	Percent
Gender	Female	1070	58.2
	Male	768	41.8
Age	under 20	80	4.4
	20–30	103	5.6
	31–40	179	9.7
	41–50	362	19.7
	51–60	518	28.2
	61–70	543	29.5
	over 70	53	2.9
Education level	Elementary school	334	18.2
	High school	380	20.7
	College	182	9.9
	University	747	40.6
	Postgraduate	195	10.6
Number of hospitalization days	3	910	49.5
	7	361	19.6
	14	368	20.0
	21	199	10.8
	Total	1838	100.0

## Results

The analyses were conducted through SPSS.19 software. All analyses were made only on complete data samples. Cases with missing values in the outcome variables were excluded from the analyses. In order to verify the fidelity of the questionnaire, we applied the method of internal consistency. Cronbach’s alpha coefficient indicates the inter-item scale composition analysis and it is based on an average of the correlations between the items of the scale. With a view to measuring the variable adequacy to the factorial model, the statistical Barlett’s sphericity test and Kaiser-Meyer-Olkin test were performed. The factorial validity was used to explore the extent to which the structure of the questionnaire is recoverable in a set of test scores. The Pearson correlation test was used to verify the existence of correlations between the studied variables. The internal consistency of the 40-item instrument was determined using Cronbach’s alpha coefficient. The obtained value of 0.72 indicated that the instrument had good reliability, thereby permitting continuation of the study. The instrument consisted of two scales: the 30-item patient dissatisfaction scale, which had a Cronbach’s alpha coefficient of 0.80, and the 10-item institutional factors scale, which had a Cronbach’s alpha coefficient of 0.60.

We also analyzed Cronbach’s alpha coefficients after systematically eliminating each item. However, the coefficients did not significantly increase after the elimination of any item. Consequently, all items measured the same construct and were retained. Regarding the construct validity, no significant differences were observed between men and women in terms of the dissatisfaction items. As can be seen in Table 2, only one item showed a statistically significant gender difference: the frequency of measuring blood pressure, t (1716.976) = 2.38, *p* = 0.01.

**Table 2:** Results of t-tests assessing sex differences in dissatisfaction item scores

Item	t	df	Sig. (2-tailed)
1.	−1.83	1676.25	0.06
2.	−1.70	1711.39	0.08
3.	−0.51	1568.01	0.60
4.	−0.94	183	0.34
5.	−2.38	1716.97	0.01
6.	−1.01	1836	0.31
7.	−1.80	1705.40	0.07
8.	−1.82	1717.79	0.06
9.	0.38	1836	0.70
10.	−1.10	1836	0.27
11.	−1.59	1667.51	0.11
12.	0.39	1717.84	0.69
13.	−2.09	1737.07	0.36
14.	1.52	1836	0.12
15.	−2.30	1777.14	0.21
16.	−2.08	1758.13	0.05
17.	0.87	1713.33	0.38
18.	−2.14	1777.01	0.32
19.	−1.93	1836	0.05
20.	2.45	1728.30	0.01
21.	1.39	1704.10	0.16
22.	−2.17	1836	0.05
23.	−1.65	1769.59	0.09
24.	2.24	1716.15	0.05
25.	−1.12	1836	0.26
26.	−1.61	1836	0.10
27.	−1.41	1836	0.15
28.	−1.26	1836	0.20
29.	1.79	1692.55	0.07
30.	0.83	1836	0.40

The mean difference of 0.41 between men and women for this item indicated that women were more dissatisfied with the procedure than were men. The item was removed from the statistical analyses at this point.

### Factor analysis of patient dissatisfaction in relation to the efficacy of the medical care received

In order to verify the factorial validity of the 30 – items’ scale, we conducted a principal component analysis with a varimax rotation. The Kaiser-Meyer-Olkin measure was used to assess if the items were suitable for the principal component analysis. As shown in Table 3, the KMO value was higher than 0.5 and Bartlett’s test of sphericity was statistically significant, indicating that principal component analysis was appropriate.

**Table 3:** Kaiser-Meyer-Olkin and Bartlett’s test

Kaiser-Meyer-Olkin Measure of Sampling Adequacy		0.64
Bartlett’s Test of Sphericity	Approx. Chi-Square	124,200.69
	df	190
	Sig.	0.00

Three latent factors corresponding to three dimensions of dissatisfaction emerged from the data: the medical staff’s ability to communicate, the medical staff’s hygiene, as well as the sanitary and privacy conditions within the hospital (see Table 4. Component matrix). The first factor explained 43.47% of the variance in patient dissatisfaction, the second factor explained 10.24% of the variance, and the third factor explained 7.59% thereof; overall, the three factors explained 61.30% of the total variance.

**Table 4:** Component matrix

Component Matrix^a^			
	Component		
	1	2	3
21. You cannot sleep	0.97		
26. You see your family and friends only for short periods of time	0.97		
20. You are disturbed by nurses/doctors when sleeping	0.97		
23. You are experiencing pain	0.96		
22. The light is on all the time	0.95		
16. You hear the sounds and alarms from medical devices and they bother you	0.95		
17. The doctors and nurses talk too loudly (noise in the department)	0.95		
11. You do not know how long you will be staying in intensive care	0.91		
12. You are restricted by tubes or perfusions	0.85		
8. The temperature in the room you are hospitalized in is too low or too high	−0.82		
7. The medical staff uses words you do not understand	−0.80		
24. You have no privacy	0.77		
9. You are disturbed by the reactions of the patients around you	−0.77		
19. You feel disoriented	0.73		
15. You feel that nurses focus more on the devices than on you	0.73		
18. You have tubes in your nose or mouth	0.72		
28. You are afraid of death	0.71		
27. You cannot communicate	0.71		
25. You hear people talking about you	0.68		
29. You are afraid of transmissible diseases	0.63		
30. Your consent for treatment was not obtained	−0.50		
4. You are aware of the smells around you		0.62	
5. You are in a mixed ward (men and women)		0.66	
6. You are seen as an object		0.59	
13. You are thirsty or hungry		0.48	
1. The procedures and treatments applied are not explained to you			0.40
3. You do not know when you are scheduled for certain procedures			0.75
2. Previously unknown members of the medical staff do not introduce themselves to patients			0.68

Extraction method: principal components analysis

^a^Three components extracted

The first factor was highly saturated, comprising 23 items, while the second factor comprised 4 items and the last factor 3 items. This first factor was a composite factor comprising three dimensions: physical discomfort, psychological comfort, and relationship with the medical staff. We concluded that this factor was valid based on its components.

The physical discomfort refers to situations like: lights turned on all the time, noise, medical staff talking about patients behind their backs, thermal comfort, difficulties in falling asleep, medical staff waking the patient up suddenly, tubes in the nose and mouth, pain experienced. Psychological discomfort includes circumstances like lack of privacy, feelings of disorientation, fear of death and transmissible diseases, inability to communicate, insufficient contact with family and friends. Relationship with medical staff includes: the feeling that nurses focus more on devices than on the patient, the medical staff uses unknown words, one’s being treated for by unknown doctors, and one’s consent for treatment is not obtained.

The second factor included items 4, 12, 17, and 21, which refer to being aware of specific hospital smells, being in a mixed ward (containing both men and women), feelings of thirst and hunger, and feeling that one is being treated as an object rather than as a human being. Although these four items appear relatively less related than those of the first factor, they all refer to the patient’s comfort or how the patient is respected and treated. The third factor comprises items 3, 9, and 14, which refer to the poor explanation of procedures and treatments by the medical staff, one’s not being informed about scheduled procedures, and members of the medical staff not introducing themselves. This factor refers to the communication abilities of the medical staff and it particularly highlights the staff’s paternalistic mentality.

In order to identify the patients’ levels of dissatisfaction with the medical staff’s professionalism, the patients’ perceptions of how they were treated during their hospitalization period were analyzed. They were asked to rank the most 5 unpleasant aspects of their hospitalization period. We included at the end of the questionnaire an item where the participants were asked to rate those first 5 items that disturbed respondents most during hospitalization.

As shown in Table 5, the five highest ranked items involved restricted movement due to medical procedures, poor communication between medical staff and patients (i.e. lack of information about procedures and consent to perform them, the staff do not relate positively to patients or are not respectful to them), and not anticipating patients’ needs. These items represent the most important reasons for patient dissatisfaction. The Pearson correlation test reveals that the older the patients are, the level of satisfaction regarding the proper care in hospital decreases (Table 6). The older they are, the outpatients consider that the level of intimacy was higher as well as the fear of the possibility of contacting diseases in hospital. However, despite the results presented above, the subjects consider that all their requests were satisfied promptly.

**Table 5:** Dissatisfaction items ranked by importance to respondents

Percent	Item
66.3	12. You are restricted by tubes or perfusions
66.2	11. You do not know how long you will be staying in intensive care
66.1	13. You are thirsty or hungry
64.4	30. Your consent for treatment was not asked for
49.1	2. Members of the medical staff do not introduce themselves
47.1	15. You feel that nurses focus more on the devices than on you
47.1	16. You hear the sounds and alarms from medical devices and they bother you
47.1	17. The doctors and nurses talk too loudly (noise in the department)
45.2	27. You cannot communicate
45.2	28. You are afraid of death
45.2	29. You are afraid of transmissible diseases
45.1	24. You have no privacy
45.1	25. You are cared for by unknown doctors
45	3. You do not know when you are scheduled for certain procedures
45	7. The medical staff uses words you do not understand
45	20. You are disturbed by nurses/doctors when sleeping
45	21. You cannot sleep
45	22. The light is on all the time
45	23. You are experiencing pain
44.8	4. You are aware of the smells around you
44.8	8. The temperature in the room you are hospitalized in is too low or too high
44.7	5. You are in a mixed ward (men and women)
44.7	6. You are seen as an object
44.7	9. You are disturbed by the reactions of the patients around you
40	1. The procedures and the treatments applied are not explained to you
31.3	18. You have tubes in your nose or mouth
31.2	19. You feel disoriented

**Table 6:** Pearson correlations coefficient obtained between Age and items no. 1, 4, 22 and 28

	Age	1.	4.	22.	28.
1.I was cared for properly	-,76^**^	1			
4.All my requests were satisfied properly	,50^*^	-,95^**^	1		
22.Do not have privacy	,70^**^	-,37^**^	,84^**^	1	
28. Are you afraid of transmissible diseases	,69^**^	-,98^**^	,70^**^	,65^**^	1

*. *p* < 0.05 (2-tailed), ** *p* < 0.01 (2-tailed), *N* = 1838

The level of education significantly correlates (Table 7) with perception of the level of pain felt and the way of granting importance. The higher the level of patients’ education is, the better the outpatients bear the pain and believe that they have been granted proper importance.

**Table 7:** Pearson correlations coefficient obtained between Level of education and items no. 3 and 7

	Level of education	3.	7.
3.I had no pain	,47^*^	1	
7.I was granted the proper importance	,53^*^	,45^**^	1

*. *p* < 0.05 (2-tailed), ** *p* < 0.01 (2-tailed), *N* = 1838

As the number of hospitalization days increases, the feeling that the nurses watch the devices more than the patients is more stronger, they feel that staff uses more specific medical terms, difficult to understand, moreover they consider the ambient temperature inadequate and feel that they were not explained the followed procedures / treatment (Table 8).

**Table 8:** Pearson correlations coefficient obtained between Number of hospitalization days and items no. 15, 7, 8 and 1

	Number of hospitalization days	15.	7.	8.	1.
15.You feel that nurses focus more on the devices than on you	-,65^**^	1			
7. The medical staff use words that you do not understand	,60^**^	-,70^**^	1		
8. The temperature in the room you are hospitalized in is too low or too high	,63^**^	-,71^**^	,99^**^	1	
1.The procedures and the treatments applied were not explained to you	-,75^**^	,37	,44	,13	1

^**^ *p* < 0.01 (2-tailed), *N* = 1838

## Discussion

This study examined first the psychometric properties and the factor structure of the new research instrument measuring patient dissatisfaction, with a sample of Romanian patients. The statistical analysis indicated that the instrument had good reliability. There were no significant differences between men and women in the responses to the dissatisfaction scale. The secondary aim was to analyze the patients’ levels of dissatisfaction with the medical staff’s professionalism. The results show that the most important reasons for patient dissatisfaction are those aspects involving restricted movement due to medical procedures, poor communication between medical staff and patients as well as the medical staff’s not anticipating patients’ needs.

The Pearson correlation test reveal that the older the patients are, the fear of contacting diseases in hospital is higher and the level of satisfaction regarding the proper care in hospital decreases. The level of education significantly and positively correlates with the perception of the level of felt pain and the way of granting importance. Less educated patients report a high level of pain bared during hospitalization and mistreatment. As the number of days of hospitalization increases, the patients tend to be more unsatisfied with the given healthcare.

### Limitations and future research

Our study is not without limitations. First, while we emphasize that the instrument is a reliable measure, we acknowledge that the statistical analysis did not include a full validation of the questionnaire. Future research should investigate the convergent and divergent validity of the measure. This is important since the patients’ dissatisfaction as a construct is understudied in Romanian context. A second limitation of the study is the inability to compare the used instrument with similar valid questionnaires. The study contributes to previous academic studies towards a better conceptual and methodological understanding of the patients’ perspective of healthcare professionalism in a Romanian context. Future studies should investigate the influence of other socio-demographical variables, which were not accounted for in this research, on patients’ perceptions of healthcare professionalism and the applicability of the instrument in more multicultural groups.

## Conclusions

The present assessment of the elements of patient dissatisfaction was not a strictly theoretical exercise; it also represents a valuable instrument for healthcare system management. Assessments of the healthcare system are essential for improving both the population’s health status and the efficacy of the healthcare system. Recent research in the field has found that patient satisfaction is influenced by the perceptions on the characteristics of the provider of services [32–34]. In line with the previous studies [35–38], this research illustrates that the outpatients’ level of dissatisfaction is influenced by the degree of attention paid by the medical staff, the communication procedures and treatments applied, the promptness to the requests of patients, and the characteristics of the physical environment.

Based on the hierarchy of factors related to patient dissatisfaction, we can conclude that in Romanian hospitals, the medical staff still adopts a paternalistic mentality. In other words, staff members do not consider important to explain procedures and treatments to the patients or the duration of these procedures, even though this would provide patients with a feeling of security. Often, medical staff members did not introduce themselves, which can strengthen the patients’ convictions that they are treated more as objects than as clients of medical services. In a review of 12,000 patient complaints, two characteristics of medical professionalism (problems in communication and perceived disrespect) were highlighted as sources of patient dissatisfaction [39].

Furthermore, many patients indicated that the hygiene within the hospitals, such as specific smells or hospitalization in mixed wards, was an issue that caused discomfort. Our study indicates that although over 25 years have passed since the regime change in Romania and the introduction of a different system of social care, there has been no corresponding change in the medical staff’s mentality or in the way patients are approached. In other words, there remains a tendency of the staff to take a paternalistic view of patients. The instrument reflects the relevant aspects of patients’ dissatisfaction. It can be used as a tool for quality improvement aims. The results are relevant in medical education, in designing effective strategies to increase outpatient satisfaction.

Patients’ satisfaction plays an important role in achieving good overall healthcare outcomes. The issue of patients’ perceptions of healthcare professionalism is important for both researchers and organizational structures. Based on the findings of this study, we suggest the improvement of the future medical professionals’ competences profile, by introducing into the curriculum, several strategies to increase the efficiency of the medical act. Some practical suggestions for policymakers include to pay attention to the quality of the systemic medical act, to make informed decisions, to build new projects to enhance the communication between medical staff and patients, to develop new evaluation standards that include the quality of interaction between patients and healthcare professionals and higher quality standards for the patients’ comfort during hospitalization period.

## Additional file

Additional file 1: Patient satisfaction questionnaire. The instrument used to asses the patient satisfaction. (DOCX 22 kb)

## Abbreviations

KMO: Kaiser-Meyer-Olkin Measure of Sampling Adequacy
