# Supplementary material for: Patients’ perceptions of healthcare professionalism—a Romanian experience
Source: BMC Health Serv Res. 2017 Jul 6;17:463. doi: 10.1186/s12913-017-2412-z (PMC5501103; doi:10.1186/s12913-017-2412-z)
Supplement: Additional file 1: — Patient satisfaction questionnaire. The instrument used to asses the patient satisfaction. (DOCX 22 kb) [file 12913_2017_2412_MOESM1_ESM.docx]

**Patient satisfaction assessment questionnaire**

We kindly ask you to participate in a quantitative research on patient satisfaction and you as the beneficiary can improve the performance of the health services. You have the right to withdraw from participation at any point during questionnaire completion, for any reason at all. The questionnaire is anonymous. Completing the survey you are giving your written consent, your agreement with the research terms and understanding of your rights as participant. Your contribution is very important and valuable in the evaluation process of these professional performances.

1. Your gender:

- Female
- Male

1. Age:

- Under 20
- 20-30
- 30-40
- 40-50
- 50-60
- 60-70
- Over 70

1. Level of education:

- Elementary education
- High school education
- College studies
- University studies
- Postgraduate studies

1. Your diagnosis

………………………………………

1. The number of days of hospitalization

- 0-3
- 4-7
- 8-14
- 15-21

1. On a scale from 1 to 10, where 1 represents the least satisfied and 10 represents extremely satisfied, how satisfied are you of the following indicators of your experience in hospital.

|  |  | 10 | 9 | 8 | 7 | 6 | 5 | 4 | 3 | 2 | 1 |
| --- | --- | --- | --- | --- | --- | --- | --- | --- | --- | --- | --- |
| 1 | You do not know how long you will be staying in intensive care. |  |  |  |  |  |  |  |  |  |  |
| 2 | You are restricted by tubes or perfusions |  |  |  |  |  |  |  |  |  |  |
| 3 | Previously unknown members of the medical staff do not introduce themselves to patients |  |  |  |  |  |  |  |  |  |  |
| 4 | You are thirsty or hungry |  |  |  |  |  |  |  |  |  |  |
| 5 | You’re blood pressure was measured too frequently |  |  |  |  |  |  |  |  |  |  |
| 6 | You feel that nurses focus more on the devices than on you |  |  |  |  |  |  |  |  |  |  |
| 7 | You hear the sounds and alarms from the medical devices and they bother you. |  |  |  |  |  |  |  |  |  |  |
| 8 | The doctors and nurses talk too loudly (noise in the department) |  |  |  |  |  |  |  |  |  |  |
| 9 | The procedures and treatments applied are not explained to you |  |  |  |  |  |  |  |  |  |  |
| 10 | You have tubes in your nose or mouth |  |  |  |  |  |  |  |  |  |  |
| 11 | You feel disoriented |  |  |  |  |  |  |  |  |  |  |
| 12 | You are in a mixed ward (men and women) |  |  |  |  |  |  |  |  |  |  |
| 13 | You see your family and friends only for short periods of time. |  |  |  |  |  |  |  |  |  |  |
| 14 | You do not know when you are scheduled for certain procedures |  |  |  |  |  |  |  |  |  |  |
| 15 | You are disturbed by nurses/doctors when sleeping |  |  |  |  |  |  |  |  |  |  |
| 16 | You cannot sleep |  |  |  |  |  |  |  |  |  |  |
| 17 | You are aware of smells around you |  |  |  |  |  |  |  |  |  |  |
| 18 | The light is on all the time |  |  |  |  |  |  |  |  |  |  |
| 19 | You are experiencing pain |  |  |  |  |  |  |  |  |  |  |
| 20 | The medical staff uses words you do not understand |  |  |  |  |  |  |  |  |  |  |
| 21 | You are seen as an object |  |  |  |  |  |  |  |  |  |  |
| 22 | You have no privacy |  |  |  |  |  |  |  |  |  |  |
| 23 | You are cared for by unknown doctors |  |  |  |  |  |  |  |  |  |  |
| 24 | The temperature in the room you are hospitalized in is too low or too high |  |  |  |  |  |  |  |  |  |  |
| 25 | You hear people talking about you |  |  |  |  |  |  |  |  |  |  |
| 26 | You cannot communicate |  |  |  |  |  |  |  |  |  |  |
| 27 | You are afraid of death |  |  |  |  |  |  |  |  |  |  |
| 28 | You are afraid of transmittable diseases |  |  |  |  |  |  |  |  |  |  |
| 29 | You are disturbed by the reactions of the patients around you |  |  |  |  |  |  |  |  |  |  |
| 30 | Your consent for treatment was not obtained |  |  |  |  |  |  |  |  |  |  |

1. Select 5 of the aspects that have bothered you the most during the hospitalization days (from point no.6.)

1.

2.

3.

4.

5.

Thank you for participating!
